# Deltamethrin Application on Pre-Weaned Calves Improves Feed Consumption, Stress and Fatigue Status under Heat Stress Conditions

**DOI:** 10.3390/pathogens11010085

**Published:** 2022-01-11

**Authors:** Konstantinos V. Arsenopoulos, Eleftherios Triantafillou, Athanasios I. Gelasakis, Elias Papadopoulos

**Affiliations:** 1 Laboratory of Parasitology and Parasitic Diseases, School of Veterinary Medicine, Faculty of Health Sciences, Aristotle University of Thessaloniki, 54124 Thessaloniki, Greece; arsenopo@vet.auth.gr; 2 “Vet Analyseis”, Veterinary Microbiological Laboratory, 41222 Larissa, Greece; eltriantafil@gmail.com; 3 Laboratory of Anatomy and Physiology of Farm Animals, Department of Animal Science, School of Animal Biosciences, Agricultural University of Athens (AUA), 11855 Athens, Greece; gelasakis@aua.gr

**Keywords:** concentrates, fatigue indicator, fly-repellent, houseflies, pre-ruminants, roughages, stress indicator, temperature-humidity index

## Abstract

Fly infestation remains a universal problem for dairy cattle herds, affecting the animals’ health and welfare status. Pre-weaned dairy calves are significantly challenged by the direct and indirect consequences of severe fly infestation, heat-stress and their interaction, which contribute to a stressful and fatiguing environment. Among several physiological, behavioral, clinical and biochemical traits, serum cortisol (SC) and creatine kinase (CK) levels, as well as feed consumption can be used as valid indicators of potential stressful and fatiguing conditions and, therefore, can be efficiently used for stress analysis studies. Hence, the objective of the study was to assess the fly-repellency effect of deltamethrin on pre-weaned dairy calves exposed to heat stress conditions, as well as its association with SC, CK concentrations and feed consumption. Two commercial dairy cattle herds of the Holstein breed in Central Macedonia (Greece) were involved in the study during summer months and under heat stress conditions. Deltamethrin administration resulted in (i) a decreased fly population (100% *Musca domestica*) landing on pre-weaned dairy calves, (ii) a reduced SC (stress indicator) and CK (fatigue indicator) concentration, and (iii) an increased consumption of feedstuff in deltamethrin treated animals compared to the untreated ones.

## 1. Introduction

In recent decades, increasing ethical concerns regarding welfare implications of livestock farming practices have reshaped farmers’ and consumers’ perceptions. The indisputable demand for the efficient assessment and protection of animal welfare has emerged and currently affects the structure and operation of modern farms. Except for the ethical perspective, appropriate welfare status provokes immunomodulatory responses, ameliorating animals’ health [1] and, consequently, reducing the use of chemical compounds for treatment (e.g., antibiotics) [2]. However, it is difficult to describe what a “sufficient” level of welfare is; therefore, the use of objective welfare indicators is needed [3,4,5,6,7].

These indicators may be direct or indirect, including physiological, behavioral, clinical and biochemical traits [1]. Among the biochemical traits, serum cortisol (SC) and creatine kinase (CK) levels can be used as indicators of potential stressful and fatiguing conditions. When animals are exposed to adverse environmental conditions or noxious stimuli (i.e., heat stress, fly annoyance, painful insect bites, etc.), the adrenal cortex is activated and the secretion of cortisol increases [1]. In addition, conditions provoking restlessness (i.e., increased body exercise, disease, discomfort, etc.) are associated with muscle cell damage and increased CK level, due to the leak of the enzyme into blood circulation [8].

Fly infestation and its efficient mitigation represent a constant challenge in dairy cattle herds [9]. This is due to the fact that flies reproduce quickly, and the time demanded for the total development from egg to adult fly may be as little as 8 days at 35 °C in cattle herds. Thereby, flies increase their numbers when hot and humid conditions prevail (i.e., during spring and summer), causing severe welfare and productivity issues in cattle herds [10,11]. Flies cause provoked annoyance, painful bites and skin lesions [12,13] and disrupt normal feeding behavior, stimulating a defensive attitude (i.e., head throwing, skin and tail twitching) [14,15] and decreasing relaxation time (i.e., fatigue). In addition, flies are carriers of pathogens, causing infectious diseases such as keratoconjunctivitis, mastitis, dermatophilosis, papillomatosis, etc. [10]

At herd level, the reduction of fly breeding areas is crucial for the prevention and control of fly infestation. This reduction can be achieved by exploiting basic hygiene practices such as draining swamps and stagnant water, the efficient management of manure and the removal of dead animals and placentas [10,16]. Additionally, the application of insecticides (specially designed for these premises) and fly repellents at the animal level may significantly contribute to the control of heavy fly infestation [17].

Several insecticidal compounds have been registered in the European Union for pour-on application in cattle. Among them, deltamethrin (a member of the pyrethroid group) is one of the most effective due to its cis-isomer form [17]. Deltamethrin expresses its repellency activity via the “hot foot effect”. In other words, flies die after the direct contact of their feet nerves with deltamethrin on the skin of the treated animal [18,19]. Currently, possible beneficial outcomes from the application of deltamethrin in the welfare status of pre-weaned dairy calves have not been assessed and sufficiently documented. The hypothesis is that application of deltamethrin in pre-weaned dairy calves reduces the rate of fly annoyance and biting and to improve the stress and fatigue status and feed the consumption of animals.

Therefore, the objective of the present study was to assess the fly-repellency effect of deltamethrin on pre-weaned dairy calves under heat stress conditions and to quantify its potential association with SC and CK concentrations and feed consumption (i.e., concentrates and roughages) in two dairy cattle farms of Greece.

## 2. Results

### 2.1. Meteorological Data

Environmental conditions, which were prevailing in both farms (farm A and B), were similar and over the typical range expected for this summer season (July and August), according to the Hellenic National Meteorological Service (Table 1). The temperature-humidity index (THI) ranged from 94 to 96 throughout the study, indicating that all pre-weaned calves experienced heat stress conditions. At the same frame, statistical analysis did not reveal significant variations of THI among sampling occasions.

### 2.2. Short and Long-Term Repellency Effect of Deltamethrin

Pour-on administration of deltamethrin (Deltanil^®^ 10 mg/mL, Virbac) was well tolerated by the calves and no local or other general adverse reactions were observed during the present study.

The effect of deltamethrin treatment on the flies’ burden, in the participating calves, is presented in Table 2. In group D, the number of flies landing on calves was reduced by 60.6 flies (*p* < 0.001, 95% CI, 57.2 to 64.0 flies) in comparison to group C (estimated marginal means were 13.6 and 74.2, for groups D and C, respectively).

### 2.3. Fly Species Identification

The common housefly (*Musca domestica*) was the only species identified in sticky traps of both farms.

### 2.4. Associations between Deltamethrin Treatment, Serum Cortisol (SC) and Creatine Kinase (CK) Concentrations

In general, mean values of SC, in pre-weaned calves of farm A and B on day 0, were 2.42 (±0.11) μg/dL and 2.46 (±0.12) μg/dL, respectively, while mean values of CK were 1199.48 (±51.59) nkat/L and 1251.06 (±41.15) nkat/L, respectively.

The effects of the deltamethrin treatment, sampling occasion, and farm on (i) the serum cortisol (SC) level and (ii) then creatine kinase (CK) level, in the studied calves are summarized in Table 2. In the group D, SC and CK concentrations were reduced by 1.86 μg/dL (*p* < 0.001, 95% CI, 1.76 to 1.97 μg/dL) and 1101 nkat/L (*p* < 0.001, 95% CI, 1009 to 1194 nkat/L) when compared to group C. The estimated marginal means of SC and CK were 1.24 and 3.10 μg/dL, and 427 and 1528 nkat/L, for groups D and C, respectively.

### 2.5. Association between Deltamethrin Treatment and Feed Consumption

Mean (±standard deviation) consumptions of concentrates in farm A were 0.51 (±0.37) kg and 0.34 (±0.29) kg, for groups D and C, respectively, while the respective values for farm B were 0.50 (±0.35) kg and 0.33 (±0.27) kg. Regarding the mean consumptions of roughages, these were 0.21 (±0.16) kg and 0.10 (±0.08) kg, for group D and C in farm A, respectively, while the respective values for farm B were 0.23 (±0.19) kg and 0.12 (±0.11) kg.

The effects of the deltamethrin treatment, sampling occasion, and farm on (i) the daily consumption of concentrates and (ii) the roughages, are summarized in Table 3. Regarding the feedstuff consumption, in group D the concentrates’ and roughages’ consumption was increased by 137 g/day (*p* < 0.001, 95% CI, 127 to 147 g/day and 25 g/day (*p* < 0.001, 95% CI, 20 to 31 g/day), respectively compared to the group C. The estimated marginal means of the daily consumption of concentrates and roughages were 566 and 429 g, and 168 and 143 g, for groups D and C, respectively.

## 3. Discussion

Heat stress is described by the temperature-humidity index (THI) value, which is a globally acceptable parameter for the quantification of its occurrence and severity. It is estimated by the combination of air temperature and relative humidity, where values up to 68 are considered as the upper critical THI of a cow’s thermoneutral zone, while values greater than 68 indicate the exposure of dairy cows to heat stress conditions [20,21]. The thermoneutral zone for pre-weaned calves is considered narrower and higher, since adult dairy cows can thermoregulate better than pre-weaned calves [22]. Even though the critical THI threshold has not been established for pre-weaned calves, it is known that the upper critical temperature is 26 °C for new-born calves and 23 °C for 1 month old calves [23]. In our study, the minimum and maximum values of the air temperature in the two farms were 37.9 °C and 39.6 °C, respectively. In addition, THI ranged from 94 to 97, which indicates that all pre-weaned calves experienced severe and similar heat stress conditions.

The air temperatures of both farms were higher (i.e., over 38.2 °C for farm A and over 37.9 °C for farm B) than the optimal range (environmental temperature between 23 °C and 27 °C and relative humidity between 65% and 90%), possibly compromising both the survival and activity of different species of flies. It is likely that under these environmental conditions, the common housefly *Musca domestica* is the most prevalent if not unique for being the only fly species surviving and being active on pre-weaned dairy calves. This is the first time this has been observed and confirms information about the housefly’s habitats and survival capability under hot and moist conditions. In any case, it could be considered an expected finding, as the optimal temperature for larval development of *Musca domestica* ranges from 30 °C to 38 °C, while as the larvae mature their temperature tolerance increases. Therefore, it is obvious that under heat stress conditions, the survival and reproduction of *Musca domestica* continues, possibly contrary to what happens to other fly species [24]. These flies cause annoyance and carry a huge variety of pathogens, including viruses, bacteria, helminths, and protozoa, as mechanical vectors actively involved in the forementioned pathogens’ transmission. Indeed, several studies have elucidated the role of *Musca domestica* in the epizootiology of mastitis, conjunctivitis and anthrax [24].

On-field reduction of a heavy fly infestation under heat stress conditions is a challenging task, and has lately emerged for the protection of calves’ welfare. For this reason, management practices, such as the removal of the manure and cleaning of premises which disrupt the flies’ habitats, are critical. In addition to management and hygiene modifications, insecticides and larvicides participate in the reduction of fly infestations [10]. Deltamethrin is a promising insecticide, which belongs to the group of synthetic pyrethroids. Although it is a commonly used drug and has been commercially available for many years [17], its efficacy against the fly infestation of pre-weaned dairy calves has not been documented. In our study, this was the first time the fly repellency effect of deltamethrin in pre-weaned dairy calves was studied, and its beneficial effects (i.e., deltamethrin treated calves had 60.6 less flies landing on their skin compared to the placebo treated ones) against heavy fly infestation were recorded. Another study, conducted by Tsoi et al. [25], confirmed the significant reduction of a heavy cat fleas (*Ctenocephalides felis*) infestation on dairy calves, after the pour-on application of deltamethrin.

Currently, the evaluation of farm animal stressors has been recognized as an important aspect for the efficient estimation of their effects on the animals’ health and welfare status [26,27]. Among the available stress indicators, SC concentration has been extensively used for the assessment of stress levels in cattle farming [28]. In this species, fly infestation has been recognized as among the factors affecting the SC level, with several studies indicating a positive relationship between cortisol concentrations and fly burdens in beef [29] and dairy cattle [30]. In dairy cattle, distress due to heavy a fly infestation deteriorates under hot-humid conditions which further increase the serum cortisol concentration [31]. Indeed, Holstein cows, kept outdoors and exposed to weather conditions, had 57% higher SC during hot-humid weather compared to indoor, housed cows [32]. This is the first study investigating the association between deltamethrin treatment and the SC of pre-weaned calves exposed to heat-stress conditions. In general, lower levels of SC were recorded in deltamethrin treated ones. It can be assumed that the application of deltamethrin reduces the population of flies, creating of a more welfare-friendly environment for pre-weaned calves when exposed to hot-humid weather. To avoid overestimation of stress levels caused by other stressors affecting overall welfare status of pre-weaned calves, such as diarrhea and pneumonia, the animals’ suffering from other welfare-challenging conditions were not selected for the study [33].

The permanent licking and biting activity of flies, such as *Musca domestica*, for extensive periods during the day is responsible for the increased annoyance which influences the overall behavior of the animals. The response to a heavy fly infestation includes defensive behaviors such as head throwing, and skin and tail twitching. These behaviors make the animals hypersensitive and contribute to decreased resting time, and increased muscular exertion and tissue damage [34,35,36]. Under these conditions, blood CK levels increase, indicating the fatigue status of the animals [8]. According to Forcados et al. [36], the CK level was significantly higher in the serum of grazing ruminants compared to non-grazing ones due to muscular exertion, suggesting damage of the muscle cells. Similarly, Earley et al. [28] suggested that CK activity was higher in bulls due to extreme restlessness during transportation. In the literature, there is little information describing the association between CK and restlessness due to fly abundance. Arsenopoulos et al. [37] recorded a lower CK level in dairy cows treated with fly repellent compared to the untreated ones. In our study, we confirmed the assumption that the consequences of a severe fly infestation on CK in pre-weaned calves can be accumulated when kept under heat-stress conditions. Under these conditions, pre-weaned calves prefer to stand for longer periods, maximizing the effective body surface area to release heat through evaporation, reducing the heat transfer from hot lying areas, and modifying the respiration rate for more efficient thermoregulation [38]. The decreased lying time subsequently increases muscle damage and fatigue and leads to higher CK concentrations in the blood. The quantification of the fly repellency effect of deltamethrin on the fatigue status of pre-weaned dairy calves suffering from heat stress is a novelty of our study.

Similar situations in the literature, where large burdens of ectoparasites such as cat fleas being responsible for discomfort and the welfare implications [39,40], were previously reported, not only in dairy calves of North America [41], Brazil [42] and Eastern Australia [25], but also in sheep and goats from Libya [43] or Israel [44].

Nutrient requirements of pre-weaned calves vary with the stage of growth. Due to the anatomical and physiological changes taking place in the gut, pre-weaned calves exclusively depend on milk or milk replacers at the early stages, and gradually adapt to solid feedstuff, known as starter feed [45]. In the studied farms, milk replacers were offered according to standard feeding protocols until weaning.

As the rumen develops, calves acquire the ability to digest both concentrate feeds and roughages to meet their nutritional demands. During artificial suckling, dry matter consumption is affected by many factors such as diseases, hygiene conditions, water supply, cold or heat stress, etc. [33,45]. Calves exposed to heat stress have a reduced average daily gain and weaning weight due to a decline in feed and nutrient intake, and an increase in water intake [46]. In addition, this welfare-challenging condition further deteriorates by fly infestation, reducing their comfort and time devoted to eating. The assumed negative association between heavy fly infestation under heat stress conditions and feed consumption of both concentrates and roughages was confirmed in our study. More precisely, pre-weaned calves exposed to heat stress with severe fly infestation reduced their daily consumption of concentrates and roughages, which corresponded to almost 0.5 Mcal of metabolizable energy, and 27.0 g of crude protein, according to their diet composition. In accordance with our study, Tsoi et al. [25] recorded an increase in feeding supported by an improved body condition score of the deltamethrin treated neonatal dairy calves against heavy cat fleas’ infestation of these animals.

## 4. Materials and Methods

### 4.1. Farms and Animals

The study was conducted in two intensively reared dairy cattle herds of a Holstein breed located at Thessaloniki (Central Macedonia, Greece). Herd A consisted of 180 lactating cows, and the routine vaccination program included a single vaccination one month before parturition for both heifers and cows against rotavirus, coronavirus and *Escherichia coli* K99 (Rotavec Corona^®^, MSD). Regarding Herd B, it consisted of 130 lactating cows and the vaccination program was the same as described for Herd A, plus a single vaccination of the heifers and cows against clostridial diseases (Cubolac^®^, Virbac) at the forementioned time-points. Animals in both herds were routinely vaccinated against bovine viral diarrhea (BVD) and infectious bovine rhinotracheitis (IBR) (Bovilis^®^, MSD) every 6 months.

In both herds, the calves were removed from their mothers immediately after their birth, were placed in individual pens and were fed with 4 to 5 L of good quality colostrum within the first 2 h *post-partum*. During the first week, all calves were fed with bulk tank milk, which was gradually replaced by a commercial milk replacer (CombiMilk^®^ Premium, Agravis) until weaning at the age of about 60 days. Concentrates were offered to the calves within the first week *post-partum*, roughages (i.e., a combination of chopped alfalfa hay and wheat straw) were added about one-month *post-partum*, while fresh clean water was provided *ad libitum*.

### 4.2. Experimental Design

The study was conducted between July and August 2020. The experimental group consisted of one hundred randomly selected pre-weaned dairy calves. Fifty calves per farm were divided in 2 similar groups (n = 25 per group) according to their age and gender. Calves of deltamethrin treated group (D group) were individually dressed once, on their back, with deltamethrin (Deltanil^®^ 10 mg/mL, Virbac Hellas, Greece) at day 15 of their age. Ten mL of the product were applied to each calf, according to the product’s instructions for application, once at minimum of every 4 weeks. On the same day (day 0), a placebo treatment was applied to the calves of the control group (C group). In all cases, deltamethrin was applied on pre-weaned dairy calves at the age of 15 days old. Different treatment groups were housed in remote areas to avoid any interactions of the fly population between groups.

### 4.3. Fly Monitoring, Trapping and Identification

Number of flies landed on each calf of both groups was recorded by direct observation of the animals. The recording protocol has been used in several studies and is considered accurate when implemented by the same observer [47,48,49]. In brief, each calf was observed by the same person from a distance of 3 to 4 m away for 2 min. Fly population was enumerated on the animal’s body from the neck to the upper part of the tail and from the spinous processes of the vertebrae to the belly and the lower legs [41]. The enumeration of the fly burden was carried out 4 more times with 10-days intervals, at days 25, 35, 45, and 55 (at about 11:00 p.m.), to assess the long-term repellency effect of deltamethrin. On day 15 an additional enumeration was done, 6 h post initial treatment, (i.e., at about 5:00 a.m.), to assess the short-term repellency effect of deltamethrin.

Moreover, a total of 10 fly traps (5 per group) with sticky surface (Fly Catcher trap, Zhejiang, China, roll of 5 × 120 cm) were set in each farm, in predefined locations, of equal distances within the pens at the level of calves. The traps were not accessible by calves and were placed at days 15, 35, and 55 after deltamethrin treatment to capture and identify fly species. The traps remained in the pens for 24 h and afterwards they were removed, put into separate plastic containers, and transferred to the Laboratory of Parasitology and Parasitic Diseases of the School of Veterinary Medicine of the Aristotle University of Thessaloniki (Greece), where they were stored in closed containers in a 70% ethanol and 30% glycerol solution until evaluated. Finally, fly species were recognized according to morphological keys, as described by Wall, Shearer [50] and Couri et al. [51], and their relevant proportion was estimated.

### 4.4. Blood Sampling and Analyses

Blood samples from each calf were collected by jugular venipuncture, in a BD Vacutainer^®^ tube without anticoagulant, at the forementioned time points of their age, i.e., days 15 (day of treatment), 25 (10 days post treatment), 35 (20 days post treatment), 45 (30 days post treatment), and 55 (40 days post treatment). Blood samples were transported to the lab within 2 h after sampling and were centrifuged at 1000 rpm for 15 min, at room temperature for the separation of the serum. Serum samples were frozen (−20 °C) until SC and CK concentration were measured. The estimation of cortisol concentration was performed with electrochemiluminescence immunoassay method (ECLIA), using Roche Cobas E601 (Diamond Diagnostics, Holliston MA, USA) immunology analyzer [52]. CK concentration was estimated using spectrophotometry (Roche Cobas C501, Diamond Diagnostics, USA) [52].

### 4.5. Quantification of Feed Consumption

Daily consumption of concentrates and roughages were estimated per calf and calculated by subtracting refusals from the offered amount after 24 h. Average daily feed intake was calculated in every sampling occasion.

Milk replacer was provided to the calves at predefined quantities according to the routine artificial suckling program of each farm. The volumes of the milk replacer provided to the pre-weaned dairy calves of both farms are present in the Table 4. Each liter of the milk replacer was prepared by the dilution of 130 g milk powder in 870 L of warm water.

### 4.6. Meteorological Data

Meteorological data for the location of each farm (Central Macedonia, Greece) were retrieved from the Hellenic National Meteorological Service database (Athens, Greece). Based on this data, temperature-humidity index (THI) was used to describe severity of heat stress the studied calves experienced. THI was estimated as follows:

THI = (1.8*T + 32) − [(0.55 − 0.0055*RH) × (1.8*T-26)]

where T = ambient temperature and RH = relative humidity.

### 4.7. Statistical Analysis

All statistical analyses were performed using SPSS v23 (IBM, New York, NY, USA) and included both descriptive (mean ± standard deviation and frequencies for continuous and categorical variables, respectively) and analytical statistics. Shapiro-Wilk and Levene’s test were used to check the assumptions of normality and homogeneity of variances, respectively, for the continuous variables. The differences of the meteorological data (i.e., air temperature, relative humidity and THI) were estimated with the chi-square test.

Dependent variables and the residuals of the initially tested linear regression models were not normally distributed; therefore, inverse Gaussian regression models were used to estimate the random effect of the s^th^ calf and the fixed effects of deltamethrin treatment, the sampling occasion, and the farm on the SC and CK levels, flies’ number, and daily consumption of concentrates and roughages, as described below:

Y_jkns_ = μ + T_j_ + S_k_ + F_n_ + γ_s_ + e_jkns_ (Model 1)

where Y_jkns_ = dependent variable (SC and CK levels, number of flies landed on a calf within a 2-min period, and daily consumption of concentrates and roughages), μ = intercept, T_j_ = fixed effect of calves group (j = 2 levels, 0 = Deltamethrin treated group, 1 = Control group), S_k_ = fixed effect of the sampling occasion (k = 5 levels, Days 15, 25, 35, 45, and 55, for daily consumption of concentrates and roughages k = 4 levels, Days 25, 35, 45 and 55), F_n_ = fixed effect of the farm (n = 2 levels, 0 = farm A, 1 = farm B), γ_s_ = repeated variation of the s^th^ calf, and e_jkns_ = residual error. In every case, first order autoregressive was used as covariance structure. Statistical significance was set at the 0.05 level.

## 5. Conclusions

Deltamethrin treatment was associated with a noticeable decrease in the number of *Musca domestica* flies, which was also the unique fly species landing on these animals under extreme hot weather conditions. The fly repellency effect of deltamethrin significantly reduced the SC and CK concentrations in treated pre-weaned dairy calves compared to the untreated ones, contributing to a more welfare-friendly environment for these animals. Increased feed consumption in pre-weaned, deltamethrin treated calves was an additional finding, which confirmed the beneficial effect of deltamethrin administration on feed efficiency and the calves’ performance during suckling under heavy fly infestation and heat stress conditions.

## Figures and Tables

**Table 1:** Air temperature (°C), relative humidity (%) and temperature-humidity index (THI) during the five sampling occasions per farm.

Farm						
Day	A			B		
	Temp. (°C)	Hum. (%)	THI	Temp. (°C)	Hum. (%)	THI
0	38.9	69	94.5	37.9	73	94.0
10	39.3	67	94.5	38.4	75	95.0
20	39.1	68	95.5	39.4	72	96.0
30	38.2	76	95.0	39.2	71	95.5
40	39.6	70	96.0	38.8	76	96.0

Temp.: Temperature, Hum.: Humidity, THI: Temperature-Humidity Index.

**Table 2:** Effects of deltamethrin treatment, sampling occasion and farm on (i) flies’ number, (ii) serum cortisol (SC) level and (iii) creatine kinase (CK) level, in the studied calves, as estimated by the inverse Gaussian mixed linear regression models applied.

					95% CI	
Parameter	Category Level	*B*	SE	*p*-Value	Lower	Upper
**Flies’ number (n)**						
Deltamethrin treatment	Yes	−60.6	1.72	0.000	−64.0	−57.2
	No	*“Ref”*				
Sampling occasion	Day 15	45.6	2.78	0.000	40.1	51.0
	Day 25	0.0	0.43	0.935	−0.8	0.9
	Day 35	−0.2	0.39	0.632	−1.0	0.6
	Day 45	0.2	0.36	0.521	−0.5	0.9
	Day 55	*“Ref”*				
Farm	A	−1.1	0.64	0.099	−2.3	0.2
	B	*“Ref”*				
**Cortisol (μg/dL)**						
Deltamethrin treatment	Yes	−1.86	0.051	0.000	−1.97	−1.76
	No	*“Ref”*				
Sampling occasion	Day 15	1.09	0.079	0.000	0.93	1.24
	Day 25	−0.13	0.048	0.008	−0.22	−0.03
	Day 35	−0.02	0.042	0.697	−0.10	0.07
	Day 45	−0.03	0.052	0.605	−0.13	0.08
	Day 55	*“Ref”*				
Farm	A	0.00	0.053	0.911	−0.11	0.10
	B	*“Ref”*				
**Creatine kinase (nkat/L)**						
Deltamethrin treatment	Yes	−1101	47.3	0.000	−1194	−1009
	No	*“Ref”*				
Sampling occasion	Day 15	828	56.3	0.000	718	939
	Day 25	−14	11.0	0.199	−36	7
	Day 35	−128	18.1	0.000	−163	−92
	Day 45	−21	9.7	0.030	−40	−2
	Day 55	*“Ref”*				
Farm	A	−15	12.6	0.248	−39	10
	B	*“Ref”*				

CI: Confidence interval (Wald confidence interval was calculated for the inverse Gaussian model); *B*: Coefficient; SE: Standard error; *“Ref”*: Reference category.

**Table 3:** Effects of deltamethrin treatment, sampling occasion and farm on (i) daily consumption of concentrates and (ii) daily consumption of roughages, in the studied calves, as estimated by the inverse Gaussian mixed linear regression models applied.

					95% CI	
Parameter	Category Level	*B*	SE	*p*-Value	Lower	Upper
**Daily consumption of concentrates (g)**						
Deltamethrin treatment	Yes	137	5.0	0.000	127	147
	No	*“Ref”*				
Sampling occasion	Day 25	−748	13.5	0.000	−775	−722
	Day 35	−570	17.0	0.000	−604	−537
	Day 45	−375	15.2	0.000	−405	−345
	Day 55	*“Ref”*				
Farm	A	−3	4.2	0.509	−11	5
	B	*“Ref”*				
**Daily consumption of roughages (g)**						
Deltamethrin treatment	Yes	25	2.8	0.000	20	31
	No	*“Ref”*				
Sampling occasion	Day 25	−314	12.7	0.000	−338	−289
	Day 35	−278	12.0	0.000	−301	−254
	Day 45	−234	9.0	0.000	−252	−216
	Day 55	*“Ref”*				
Farm	A	6	3.1	0.073	−1	12
	B	*“Ref”*				

CI: Confidence interval (Wald confidence interval was calculated for the inverse Gaussian model); *B*: Coefficient; SE: Standard error; *“Ref”*: Reference category.

**Table 4:** Feeding program of milk/milk replacer in both farms.

Week	Milk/MR (L) (Divided in 2 Meals per Day)
1st	4.5–6
2nd	7
3rd	7
4th	7
5th	7
6th	7
7th	5
8th	2
9th	0

MR: milk replacer.

## Data Availability

The data presented in this study are available in the main text, figures, and tables.

## References

[1] Moberg G.P., Moberg G.P., Mench J.A. (2009). Biological response to stress: Implications for animal welfare. The Biology of Animal Stress: Basic Principles and Implications for Animal Welfare.

[2] Poveda J.M., Jimnez L., Perea J.M., Arias R., Palop M.L. (2020). Farming Practices Influence Antibiotic Resistance and Biogenic Amine Capacity of Staphylococci from Bulk Tank Ewe’s Milk. Animals.

[3] Hemsworth P.H., Barnett J.L., Beveridge L., Matthews L.R. (1995). The welfare of extensively managed dairy cattle: A review. Appl. Anim. Behav. Sci..

[4] Sybesma W. (1981). The Welfare of Pigs.

[5] Smidt D. (1983). Indicators Relevant to Farm Animal Welfare.

[6] Baxter S.H., Baxter M.R., MacCormack J.A.C. (1983). Farm Animal Housing and Welfare.

[7] Zayan R., Dantzer R. (1990). Social Stress in Domestic Animals.

[8] Brancaccio P., Lippi G., Maffulli N. (2010). Biochemical markers of muscular damage. Clin. Chem. Lab. Med..

[9] Hillerton J.E., Bramley A.J., Yarrow N.H. (1985). Control of flies (Diptera: Muscidae) on dairy heifers by electron ear-tags. Br. Vet. J..

[10] Scott D.W., Divers T., Peck S. (2008). Skin diseases. Rebhun’s Diseases of Dairy Cattle.

[11] Taylor D.B., Moon R.D., Mark D.R. (2012). Economic impact of stable flies (Diptera: Muscidae) on dairy and beef cattle production. J. Med. Entomol..

[12] Vitela-Mendoza I., Cruz-Vazquez C., Orihuela A. (2006). A note on the effect of controlling stable flies (*Stomoxys calcitrans*) in the resting activity and pen distribution of dairy cows. J. Appl. Anim. Welf. Sci..

[13] Vitela-Mendoza I., Cruz-Vazquez C., Solano J.J., Orihuela A. (2007). A note on the associations between the prevalence of stable flies (*Stomoxys calcitrans*) and the behaviour of dairy cows under semi-arid conditions. J. Anim. Vet. Adv..

[14] Dougherty C.T., Knapp F.W., Burrus P.B., Willis D.C., Cornelius P.L., Dougherty N.W., Knapp F.W., Burrus P.B., Willis D.C., Cornelius P.L. (1994). Moderation of grazing behavior of beef cattle by stable flies (*Stomoxys calcitrans L.*). Appl. Anim. Behav. Sci..

[15] Mullens B.A., Lii K.S., Mao Y., Meyer J.A., Peterson N.G., Szijj C.E. (2006). Behavioral responses of dairy cattle to the stable fly, *Stomoxys calcitrans*, in an open field environment. Med. Vet. Entomol..

[16] Taylor D.B., Berkebile D.R. (2011). Phenology of stable fly (Diptera: Muscidae) larvae in round bale hay feeding sites in eastern Nebraska. Environ. Entomol..

[17] Mehlhorn H., Al-Rasheid K.A.S., Abdel-Ghaffar F., Klimpel S., Pohle H. (2010). Life cycle and attacks of ectoparasites on ruminants during the year in Central Europe: Recommendations for treatment with insecticides (e.g., Butox^®^). Parasitol. Res..

[18] Eckert J., Friedhoff K.T., Zahner H., Deplazes P. (2009). Lehrbuch der Parasitologie für die Tiermedizin.

[19] Dettner K., Peters W. (2010). Lehrbuch der Entomologie.

[20] Collier R.J., Zimbelman R.B., Rhoads R.P., Rhoads M.L., Baumgard L.H. A re-evaluation of the impact of temperature humidity index (THI) and black globe humidity index (BGHI) on milk production in high producing dairy cows. Proceedings of the Southwest Nutritional Management Conference.

[21] Young H., Parchment B., Ayala A.L., Progar A.A. (2020). Physiological responses of Holstein calves to hot weather conditions. Int. J. Biometeorol..

[22] Bateman H.G., Hill T.M., Aldrich J.M., Schlotterbeck R.L., Firkins J.L. (2012). Meta-analysis of the impact of initial serum protein concentration and empirical prediction model for growth of neonatal Holstein calves through eight weeks of age. J. Dairy Sci..

[23] Wathes C.M., Jones C., Webster A. (1983). Ventilation, air hygiene and animal health. Vet. Rec..

[24] Taylor M.A., Coop R.L., Wall R.L., Taylor M.A., Coop R.L., Wall R.L. (2007). Facultative ectoparasites and arthropod vectors. Veterinary Parasitology.

[25] Tsoi F.M.B., Slapeta J., Reynolds M. (2020). *Ctenocephalides felis* (cat flea) infestation in neonatal dairy calves managed with deltamethrin pour-on in Australia. Vet. Parasitol..

[26] Mοstl E., Palme R. (2002). Hormones as indicators of stress. Domest. Anim. Endocrinol..

[27] Saco Y., Fina M., Gimenez M., Pato R., Piedrafita J., Bassols A. (2008). Evaluation of serum cortisol, metabolic parameters, acute phase proteins and faecal corticosterone as indicators of stress in cows. Vet. J..

[28] Earley B., McDonnell B., Murray M., Prendiville D., Crowe M. (2011). The effect of sea transport from Ireland to the Lebanon on inflammatory, adrenocortical, metabolic and behavioural responses of bulls. Res. Veter. Sci..

[29] Schwinghammer K.A., Knapp F.W., Boling J.A., Schillo K.K. (1986). Physiological and nutritional response of beef steers to infestations of the stable fly (Diptera: Muscidae). J. Econ. Entomol..

[30] Vitela-Mendoza I., Cruz-Vazquez C., Solano-Vergara J., Orihuela-Trujillo A. (2016). Short communication: Relationship between serum cortisol concentration and defensive behavioral responses of dairy cows exposed to natural infestation by stable fly, *Stomoxys calcitrans*. J. Dairy Sci..

[31] Chen S., Wang J., Peng D., Li G., Chen J., Gu X. (2018). Exposure to heat stress environment affects the physiology, circulation levels of cytokines and microbiome in dairy cows. Sci. Rep..

[32] Wise M.E., Armstrong D.V., Huber J.T., Hunter R., Wiersma F. (1988). Hormonal alterations in the lactating dairy cow in response to thermal stress. J. Dairy Sci..

[33] Cohen S., Janicki-Deverts D., Doyle W.J., Miller G.E., Frank E., Rabin B.S., Turner R.B. (2012). Chronic stress, glucocorticoid receptor resistance, inflammation and disease risk. Proc. Natl. Acad. Sci. USA.

[34] Heffron J., Mitchell G., Dreyer J. (1982). Muscle fibre type, fibre diameter and pH_1_ values of *M. longissimus dorsi* of normal, malignant hyperthermia and pse-susceptible pigs. Br. Veter. J..

[35] Mitchell G., Hattingh J., Ganhao M. (1988). Stress in cattle assessed after handling, after transport and after slaughter. Vet. Rec..

[36] Forcados G.E., Lohlum A.S., Usman Y., Tondo B.K., Atiku A.A. (2016). Changes in serum biochemical parameters and oxidative stress biomarkers in grazing cattle. Comp. Haematol. Int..

[37] Arsenopoulos K., Triantafillou E., Papadopoulos E. (2017). Reduced stress and fatigue indicators (cortisol and creatine kinase) in dairy cattle due to fly repellency using deltamethrin (Butox^®^, MSD). Bulg. J. Vet. Med..

[38] Kim W.S., Lee J.S., Jeon S.W., Peng D.Q., Kim Y.S., Bae M.H., Jo Y.H., Lee H.G. (2018). Correlation between blood, physiological and behavioral parameters in beef calves under heat stress. Asian-Australas. J. Anim. Sci..

[39] Halos L., Beugnet F., Cardoso L., Farkas R., Franc M., Guillot J., Pfister K., Wall R. (2014). Flea control failure? Myths and realities. Trends Parasitol..

[40] Rust K.M. (2017). The biology and ecology of cat fleas and advancements in their pest management: A review. Insects.

[41] Dryden M.W., Broce A.B., Moore W.E. (1993). Severe flea infestation in dairy calves. J. Am. Vet. Med. Assoc..

[42] Araujo F.R., Silva M.P., Lopes A.A., Ribeiro O.C., Pires P.P., Carvalho C.M., Balbuena C.B., Villas A.A., Ramos J. (1998). Severe cat flea infestation of dairy calves in Brazil. Vet. Parasitol..

[43] Kaal J.F., Baker K., Torgerson P.R. (2006). Epidemiology of flea infestation of ruminants in Libya. Vet. Parasitol..

[44] Yeruham I., Rosen S., Hadani A. (1989). Mortality in calves, lambs and kids caused by severe infestation with the cat flea *Ctenocephalides felis felis* (Bouche, 1835) in Israel. Vet. Parasitol..

[45] Krishnamoorthy U., Moran J. (2011). Rearing Young Ruminants on Milk Replacers and Starter Feeds.

[46] Dado-Senn B., Acosta L.V., Rivera M.T., Field S.L., Marrero M.G., Davidson B.D., Tao S., Fabris T.F., Ortiz-Colon G., Dahl G.E. (2020). Pre- and postnatal heat stress abatement affects dairy calf thermoregulation and performance. J. Dairy Sci..

[47] Castro E., Gil A., Solari M.A., Farias N.A. (2005). Validation of a subjective counting method for a horn flies (*Haematobia irritans irritans*) (Diptera: Muscidae) population in a cattle herd. Vet. Parasitol..

[48] Mullens B.A., Soto D., Gerry A.C. (2016). Estimating field densities of *Haematobia irritans* (Diptera: Muscidae) using direct visual field counts versus photographic assessments. J. Med.  Entomol..

[49] Mullens B.A., Watson D.W., Gerry A.C., Sandelin B.A., Soto D., Rawls D., Denning S., Guisewite L., Cammack J. (2017). Field trials of fatty acids and geraniol applied to cattle for suppression of horn flies, *Haematobia irritans* (Diptera: Muscidae), with observations on fly defensive behaviors. Vet. Parasitol..

[50] Wall R., Shearer D., Wall R., Shearer D. (2001). Adult Flies (Diptera). Veterinary Ectoparasites: Biology, Pathology and Control.

[51] Couri M.S., Pont A.C., Penny N.D. (2006). Muscidae (Diptera) from Madagascar: Identification keys, descriptions of new species and new records. Proc. Calif. Acad. Sci..

[52] Cobas^®^ 6000 Analyzer Series. https://diagnostics.roche.com/global/en/products/systems/cobas_-6000-analyzer-series.html.

